# The epidemiological trends in the burden of lung cancer attributable to PM_2.5_ exposure in China

**DOI:** 10.1186/s12889-021-10765-1

**Published:** 2021-04-15

**Authors:** Xiaomei Wu, Bo Zhu, Jin Zhou, Yifei Bi, Shuang Xu, Baosen Zhou

**Affiliations:** 1 Department of Clinical Epidemiology and Center of Evidence Based Medicine, The First Hospital of China Medical University, No. 155 Nanjing Bei Street, Heping District, Shenyang, 110001 Liaoning Province China; 2 Department of Cancer Prevention and Treatment, Cancer Hospital of China Medical University/Liaoning Cancer Hospital & Institute, No. 44 Xiaoheyan Road, Dadong District, Shenyang, 110001 Liaoning Province China; 3 Department of Medical Oncology, Cancer Hospital of China Medical University/Liaoning Cancer Hospital & Institute, Shenyang, Liaoning Province China; 4 College of Foreign Languages, University of Shanghai for Science and Technology, Shanghai, China; 5 Library of China Medical University, Shenyang, Liaoning Province China

**Keywords:** PM_2.5_, Ambient exposure, Household exposure, Lung cancer, Age-period-cohort analysis

## Abstract

**Objective:**

PM_2.5_, which is a major contributor to air pollution, has large effects on lung cancer mortality. We want to analyse the long-term trends in lung cancer burden attributable to PM_2.5_ exposure and provide evidence that can be used for preventive measures and health resource planning.

**Methods:**

Mortality data related to lung cancer were obtained from the Global Burden of Disease (GBD) 2019 project. A joinpoint regression analysis was used to assess the magnitude and direction of the trends in mortality from 1990 to 2019, and the age-period-cohort method was used to analyse the temporal trends in the mortality rate of lung cancer attributable to PM_2.5_ exposure by age, period, and cohort.

**Results:**

From 1990 to 2019, the age-standardized mortality rate (ASMR) attributable to PM_2.5_ exposure trended slowly upwards, and the ASMR due to ambient PM_2.5_ exposure (APE) increased significantly, that due to household PM_2.5_ exposure (HPE) decreased. The longitudinal age curves show that the mortality rates due to PM_2.5_ exposure among younger individuals were low, and they significantly increased from their levels among those in the 45–49 age group to their levels among those in the over-85 age group. From 1990 to 2019, the period RRs due to APE increased, but those due to HPE decreased. Similar trends were observed in the cohort RRs. The overall net drift per year attributable to PM_2.5_ exposure was below 0. The local drift values increased with age and were above 0 for the over-80 age groups. The overall net drifts per year were above zero for APE and below zero for HPE. The corresponding results among males were higher than those among females.

**Conclusions:**

In China, the type of air pollution responsible for lung cancer has changed from household air pollution to ambient air pollution. PM_2.5_ exposure is more harmful among males and older people. Ambient air pollution should be emphasized, and China should strengthen its implementation of effective public policies and other interventions.

**Supplementary Information:**

The online version contains supplementary material available at 10.1186/s12889-021-10765-1.

## Introduction

Air pollution is an important global health problem, and the severity of air pollution in China has attracted attention worldwide [1]. Air pollution refers to both ambient and household air pollution. Ambient air pollution mainly comes from traffic, factories and household fuel, and household air pollution mainly comes from cooking and heating biomass and coal fuel [2]. As the most widely studied air pollutant, PM_2.5_ is increasingly used as an indicator of pollution, with annual average concentrations ranging from less than 10 to more than 100 μg/m^3^ globally; it is associated with the risk of many noncommunicable diseases, such as cardiovascular disease [3], chronic obstructive pulmonary disease (COPD) [4] and diabetes [5], and it led to 8.3 million premature deaths in 2017. After evaluating this component of air pollution, the International Agency for Research on Cancer (IARC) unanimously agreed that PM_2.5_ is carcinogenic to humans (Group 1 carcinogen) [2].

A growing number of studies have shown that PM_2.5_ exposure is closely related to the risk of lung cancer. In a large cohort study on long-term ambient PM_2.5_ exposure (APE) in Canada, Bai et al. found that each 5 μg/m^3^ increase in the ambient PM_2.5_ concentration was associated with a 2% (95% CI: 1–5%) increase in the risk of lung cancer after adjusting for a series of individual and area-level risk factors [6]. In the AHSMOG-2 Study, Gharibvand L et al. also found that each 10-μg/m^3^ increase in ambient PM_2.5_ concentration was associated with a 43% (95% CI: 11–84%) increase in the risk of lung cancer [7]. In the European Study of Cohorts for Air Pollution Effects, data from 17 cohort studies based in nine European countries were used, and the results also showed a statistically significant association between the risk for adenocarcinomas of the lung and ambient PM_2.5_ [8]. Household PM_2.5_ exposure (HPE) is an important component of household air pollution. Approximately 17% of lung cancer deaths in adults are attributable to exposure to carcinogens via household air pollution caused by cooking with kerosene or solid fuels such as wood, charcoal or coal. The estimated number of lung cancer deaths attributable to HPE in China was 271,089 in 2017, which was the largest number in the world [9].

In China, lung cancer ranked as the 20th leading cause of human death in 1990, had increased to the 14th leading cause of death by 2019, and is the leading cause of cancer death among both men and women. More than one-third of all newly diagnosed lung cancers occur in China [10]. Lung cancer has imposed enormous health and economic burdens on patients, families and the whole country. Air pollution in China has become a serious environmental problem and has attracted increasing attention. At present, there are several valuable studies exploring the relationship between air pollution or PM_2.5_ in China and disease burdens [11–14]. However, there are few studies on its relation to the burden of lung cancer [15], especially in terms of long-term trends. Therefore, it is very important to evaluate the share of the lung cancer burden caused by air pollution, especially in PM_2.5_. Therefore, in our study, we want to analyse the changes in the share of the lung cancer burden attributable to PM_2.5_ exposure from 1990 to 2019. Moreover, there have been few studies that comprehensively analyse the possible mechanisms underlying the long-term trends in the lung cancer burden attributable to PM_2.5_ exposure. We estimated an age-period-cohort model to analyse the independent effects of chronological age, time period, and birth cohort and provide a theoretical basis for public health policy related to PM_2.5_-induced health effects.

## Materials and methods

### Data source

The mortality rate of lung cancer (C33–C34), as identified by the International Classification of Diseases (ICD) version 10 (ICD-10), from 1990 to 2019 in China was extracted from the Global Burden of Disease (GBD) 2019 project, which is available at the GBD Data Tool repository and can be accessed at http://ghdx.healthdata.org/gbd-results-tool. To date, GBD 2019 is the most comprehensive effort to measure epidemiological levels and trends worldwide; contains data on 84 behavioural, environmental, occupational, and metabolic risks or clusters of risks, 282 causes of death, 359 diseases and injuries and the healthy life expectancy (HALE) for 195 countries and territories; and quantifies the impact of hundreds of diseases, injuries, and risk factors in countries around the world. PM_2.5_ exposure includes both APE and HPE. APE is defined as annual average daily exposure to outdoor air concentrations of PM_2.5_, and HPE is defined as individual exposure to PM_2.5_ due to the use of solid cooking fuel. The mortality rates were age-standardized according to the GBD 2019 global age-standardized population. We added the details about how the data was extracted from the GBD 2019 in the Supplementary 1.

### Statistical analyses

The relevant methods for mortality standardization in the GBD database have been introduced in previous studies [16, 17]. To assess the magnitude and direction of trends in the mortality rate of lung cancer over time, we used JoinPoint software (Version 4.7.0.0) to calculate the average annual percentage change (AAPC) and the corresponding 95% CIs by joinpoint regression analysis. The JoinPoint software took the trend data and fitted the simplest possible joinpoint model to the data, with the natural logarithm of the age-standardized mortality rates as the dependent variable and the calendar year as the independent variable. The significance tests used a Monte Carlo permutation method, and the overall asymptotic significance level was obtained through a Bonferroni correction.

To assess the mortality rate of lung cancer in the population in a particular year and the accumulation of health risks since birth, we used an age-period-cohort model to analyse the temporal trends in lung cancer mortality rate attributable to PM_2.5_ exposure by age, period, and cohort. The age-period-cohort model provides a useful parametric framework that complements standard nonparametric descriptive methods. The longitudinal age curve represents the fitted longitudinal age-specific rates relative to the reference cohorts adjusted for period deviations. The age effect refers to age-related physiological and pathological changes that affect disease mortality rates. The period rate ratios (period RRs) are the ratios of age-specific rates in a given period compared to the reference period. The period effect refers to changes in disease mortality rate caused by various events over time, such as the introduction of effective treatments, the implementation of screening procedures, and the increasingly stringent air pollution control policies introduced by the Chinese government over the past 30 years. The cohort rate ratios (cohort RRs) are the ratios of age-specific rates in a given cohort compared to the reference cohort. The cohort effects refer to differences in disease mortality rates between generations as a consequence of lifestyle changes over time or different exposure to risk factors, such as through changes in dietary structures, cooking habits, or living and kitchen environments. Local drifts represent the annual percentage change in the expected age-specific rates over time. Net drift represents the annual percentage change in the expected age-adjusted rates over time. And we added the definitions of APC model parameters in the Supplementary 2.

In our model, we needed to convert the collected data into successive 5-year age groups and consecutive 5-year periods. Because the GBD dataset does not provide successive 5-year age groups for those over 85 years old in the related mortality data on lung cancer attributable to PM_2.5_ exposure and the ASMRs of lung cancer attributable to PM_2.5_ for those under the age of 24 in GBD are equal to 0, the related mortality data on lung cancer attributable to PM_2.5_ exposure were recoded into successive 5-year age groups for those aged from 25 to 29 years to 80–84 years and one group for those aged over 85 years and consecutive 5-year periods (1990–1994 to 2015–2019). A general linear model was used to analyse the slope of the period/cohort RRs. The statistical analysis was performed by R statistical software (R version 3.5.1), and *p* < 0.05 was considered significant.

## Results

### The temporal trend in the age-standardized mortality rate (ASMR) of lung cancer attributable to PM_2.5_ exposure from 1990 to 2019

**Fig. 1:**
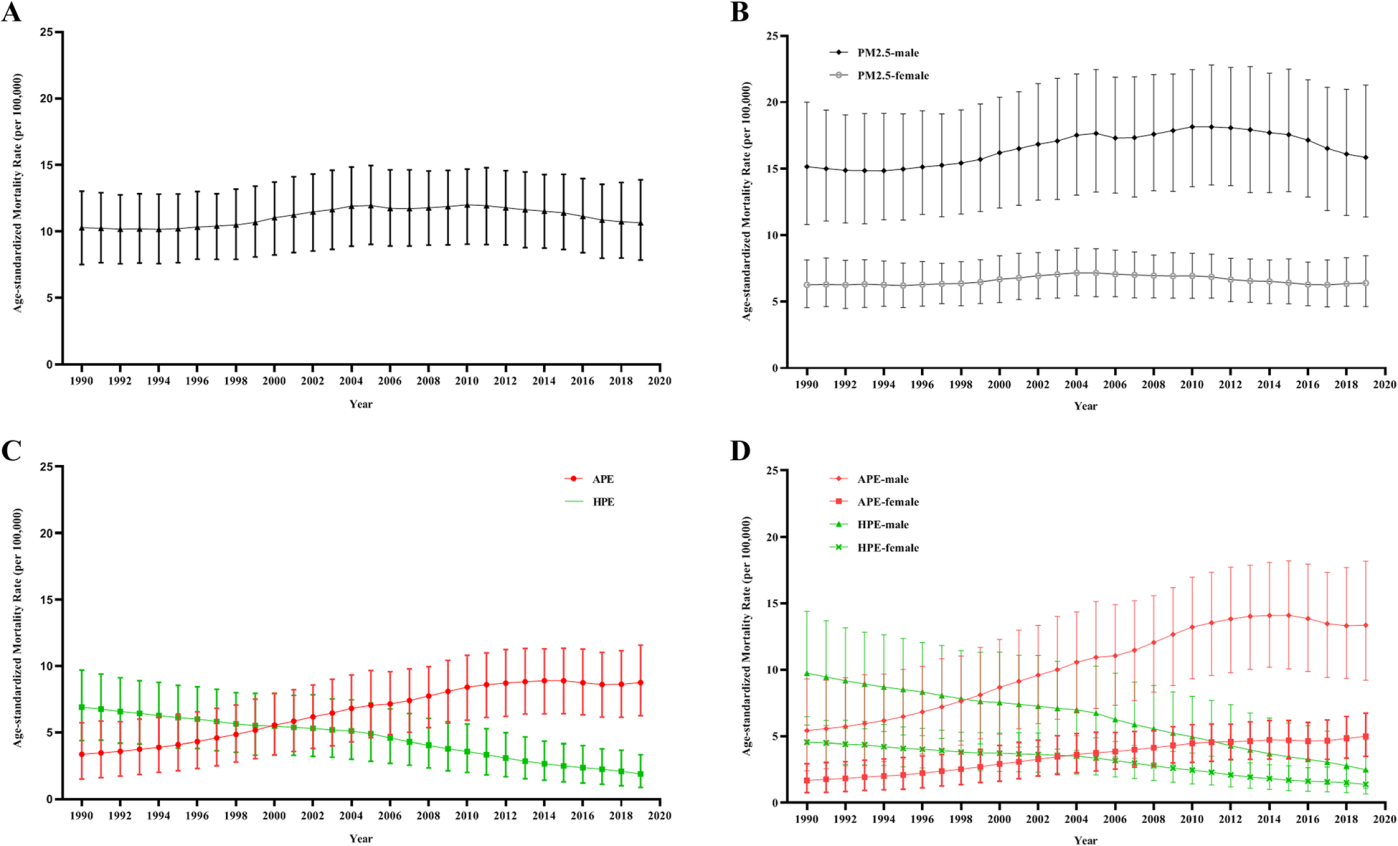
The temporal trends in the ASMR of lung cancer attributable to PM_2.5_ exposure from 1990 to 2019. **a** The mortality rate for both sexes due to PM_2.5_ exposure. **b** The mortality rate due to PM_2.5_ exposure by sex. **c** The mortality rate due to APE and HPE for both sexes. **d** The mortality rate due to APE and HPE by sex

Figure 1 shows the temporal trend in the ASMR of lung cancer attributable to PM_2.5_ exposure from 1990 to 2019. Table 1 shows the results of the joinpoint regression analysis on the ASMR of lung cancer attributable to PM_2.5_ exposure from 1990 to 2019. Over the past 30 years, the number of deaths increased 2.5 times over, from 84,434 in 1990 to 208,587 in 2019. The ASMR increased slightly among males but not among females. We further analysed the ASMRs of lung cancer attributable to APE and HPE. For APE, the number of deaths increased 6.2 times over, from 27,637 in 1990 to 171,300 in 2019. The ASMR increased significantly among both sexes (AAPC: 3.8%; 95% CI: 3.4, 4.2%), that is, among both males and females. Compared to that attributable to APE, the ASMR of lung cancer attributable to HPE decreased significantly among both sexes (AAPC: − 4.2%; 95% CI: − 4.6, − 3.8%), that is, among both males and females. The ASMR due to APE was higher than that due to HPE after 2001. For males, the ASMR due to APE was higher than that due to HPE after 1998, and for females, the ASMR due to APE was higher than that due to HPE after 2005. (Table 1 and Fig. 1).

**Table 1:** The results by joinpoint regression analysis on the mortality rate of lung cancer attributable to PM2.5 exposure from 1990 to 2019

	PM		APE		HPE	
	AAPC(%)	95%CI(%)	AAPC (%)	95%CI (%)	AAPC (%)	95%CI(%)
**Both sexes**	0.4^a^	(0.2, 0.6)	3.8^a^	(3.4, 4.2)	−4.2^a^	(−4.6, −3.8)
**Male**	0.6^a^	(0.4, 0.8)	3.7^a^	(3.3, 4.1)	−4.3^a^	(−4.7, −3.9)
**Female**	0.1	(−0.1, 0.3)	4.0^a^	(3.6, 4.4)	−4.1^a^	(−4.5, −3.6)

^a^statistically significant (*p* < 0.05); *AAPC* average annual percent change

### The age-period-cohort analysis of the mortality rate of lung cancer attributable to PM_2.5_ exposure from 1990 to 2019

**Fig. 2:**
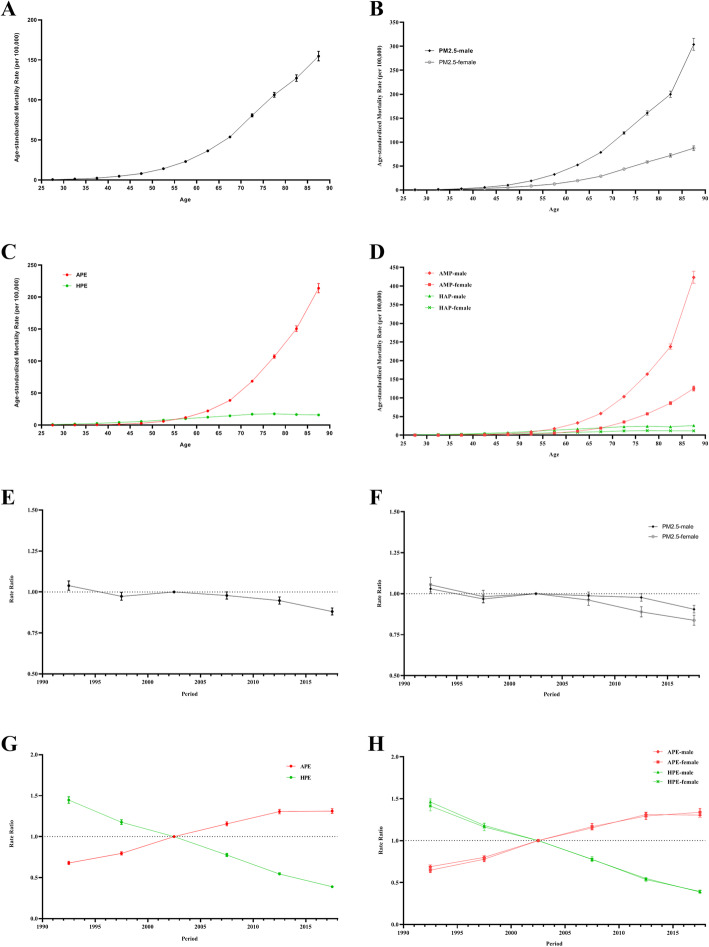
The longitudinal age curves and period RRs of the mortality rate of lung cancer attributable to PM_2.5_ exposure from 1990 to 2019. **a** The longitudinal age curves for both sexes in PM_2.5_ exposure. **b** The longitudinal age curves by sex in PM_2.5_ exposure. **c** The longitudinal age curves for both sexes in APE and HPE. **d** The longitudinal age curves by sex in APE and HPE. **e** The period RRs for both sexes in PM_2.5_ exposure. **f** The period RRs by sex in PM_2.5_ exposure. **g** The period RRs for both sexes in APE and HPE. **h** The period RRs by sex in APE and HPE

**Fig. 3:**
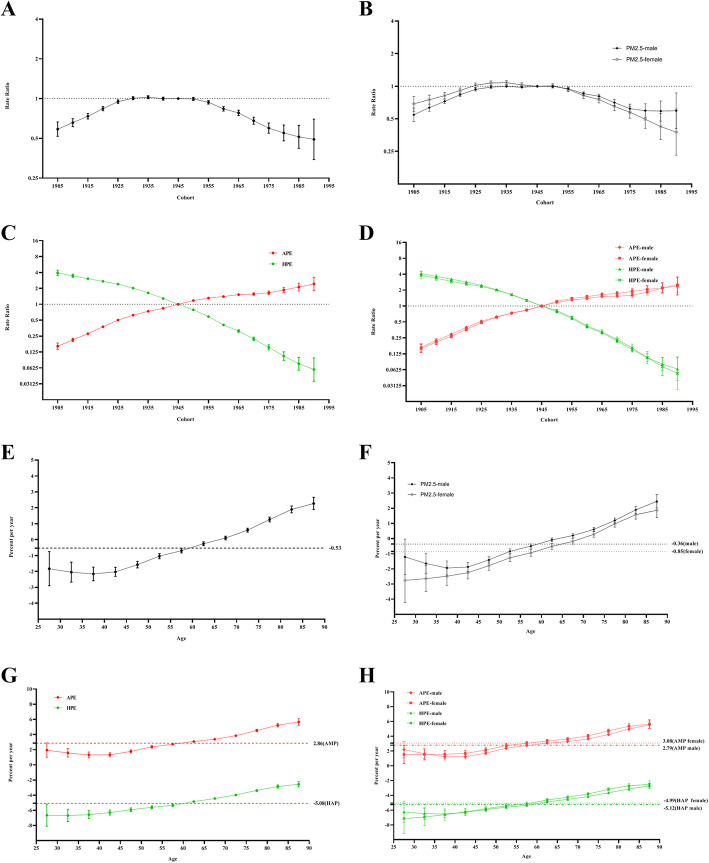
The cohort RRs and local drifts of the mortality rate of lung cancer attributable to PM_2.5_ exposure from 1990 to 2019. **a** The cohort RRs for both sexes in PM_2.5_ exposure. **b** The cohort RRs by sex in PM_2.5_ exposure. **c** The cohort RRs for both sexes in APE and HPE. **d** The cohort RRs by sex in APE and HPE. **e** The local drifts for both sexes in PM_2.5_ exposure. **f** The local drifts by sex in PM_2.5_ exposure. **g** The local drifts for both sexes in APE and HPE. **h** The local drifts by sex in APE and HPE

For the same birth cohort, the mortality rate attributable to PM_2.5_ exposure increased with age, and the mortality rate among males was higher than that among females in each age group (Fig. 2a and b). In terms of APE and HPE, the mortality rates due to APE were lower than those due to HPE for the age groups < 55 years but higher than those due to HPE for the 55 or older age groups, and they significantly increased with age among those older groups. Similar changes were observed in males and females (Fig. 2c and d). The period RRs attributable to PM_2.5_ exposure trended slowly downwards, and the downward trend among females was steeper than that among males (Fig. 2e and f). In terms of APE and HPE, the period RRs for APE increased from 1990 to 2019, but the period RRs for HPE decreased from 1990 to 2019. This occurred for both males and females, meaning there was no difference between males and females (Fig. 2g and h). The cohort RRs attributable to PM_2.5_ exposure increased among those born before 1930 and then gradually decreased among those born after 1930. Similar trends were observed among both males and females (Fig. 3a and b). The cohort RRs for APE trended upwards among those born from 1905 to 1990, while for HPE, they trended downwards (Fig. 3c and d). The overall net drift value attributable to PM_2.5_ exposure per year was − 0.53% (95% CI, − 0.65% to − 0.49%). The local drift values increased by age group and were above 0 in the over-80 age groups among both males and females (Fig. 3e and f). The overall net drifts per year were 2.85% (95% CI, 2.74 to 2.97%) for APE and − 5.08% (95% CI, − 5.22 to − 4.94%) for HPE. The local drift values for APE and HPE also increased by age group. All the local drift values were above 0 for APE and below 0 for HPE. Similar changes were observed among both males and females (Fig. 3g and h). Figures 2 and 3 show the detailed results of the age-period-cohort analysis of the mortality rate of lung cancer attributable to PM_2.5_ exposure from 1990 to 2019. The age, period and cohort effect were statistically significant, and the detailed results were shown in Supplementary 3.

## Discussion

Over the past 30 years, China has experienced rapid industrialization, urbanization and urban transport development, and increases in industrial emissions, urban construction and vehicle exhaust have caused serious air pollution. In recent years, studies on air pollution have increased and have found that PM_2.5_ exposure is associated with all-cause, lung cancer, and cardiopulmonary mortality. Fine particulate air pollution is associated with an approximately 4, 6, and 8% increase in the risk of all-cause, cardiopulmonary, and lung cancer mortality, respectively [18]. The study areas have included Beijing [12], Shanghai [12], Guangzhou [13], Taiyuan [11], Shenyang [14] and other large cities in China. Compared with that in developed countries, the type of air pollution in Chinese cities changed from traditional soot pollution to hybrid soot/vehicle exhaust pollution, leading to complex and diverse sources of particulate matter [19]. As PM_2.5_ levels have increased, the related health effects and disease burdens have received more attention. An increase of 10 μg/m^3^ of PM_2.5_ is associated with a 12% increase in the risk of mortality due to lung cancer, and the concentration response curve suggests a nonlinear relationship between PM_2.5_ and mortality in China, where PM_2.5_ exposure is higher than in developed countries [15, 20]. Therefore, research on the association between PM_2.5_ and lung cancer is very important and necessary, and is helpful in clarifying the impact of PM_2.5_ on lung cancer in China and for formulating targeted measures to reduce the disease burden.

In our study of lung cancer cases attributable to PM_2.5_, from 1990 to 2019, the number of lung cancer deaths attributable to APE accounted for the majority of all lung cancer deaths. Regarding the mortality rates, the ASMRs due to APE increased significantly from 1990 to 2019, while those due to HPE decreased significantly. After 2010, the ASMRs due to APE were significantly higher than those due to HPE. For both APE and HPE, the ASMRs among males were higher than those among females, and after 2009, the ASMRs due to APE among males were the highest. This shows that PM_2.5_ exposure in China has shifted from indoor pollution to outdoor pollution. This may be due to China’s economic development; the use of central heating, gas and range hoods has greatly reduced indoor PM_2.5_ production, while China’s extensive use of coal and other energy sources has led to the aggravation of outdoor air pollution [21, 22]. We further analysed the effects of age, period and cohort on the epidemiological changes in lung cancer attributable to PM_2.5_ exposure. In the longitudinal age curves, the mortality rate of lung cancer attributable to PM_2.5_ exposure was low among the younger age groups, and ASMRs due to both APE and HPE significantly increased from their levels among those 45–49 years to their levels among those over 85 years. This may be mainly related to immune system decline in older people. Therefore, policy actions to reduce the lung cancer burden attributable to PM_2.5_ exposure should focus on controlling the outdoor PM_2.5_ concentrations to which elderly individuals are exposed.

Unlike the slow downward trend in the period RRs attributable to PM_2.5_ exposure, the period effects were opposites for APE and HPE: for APE, the period effects increased, but for HPE, they declined. Regarding HPE, China has always attached great importance to reducing indoor and outdoor air pollution. Since the early 1980s, China has introduced more than 180 million improved stoves to improve household energy use. All introduced stoves have chimneys, and some have manual or electric blowers to promote more efficient combustion in order to reduce the concentration of indoor pollutants [21]. Regarding APE, since the beginning of the twenty-first century, given the serious ambient air pollution problem in China, the government has implemented emission-control policies that have been continuously tightened since 2005, and the overarching goal has been to reduce total emissions of air pollutants. In 2013, the government issued the “Air Pollution Prevention and Control Action Plan”, which is the most stringent policy on air pollution in Chinese history [23]. Additionally, in 2018, the government issued a three-year action plan to win the “blue sky defence war”, a proposal to effectively promote clean heating in the northern region, accelerate the transition from coal to electricity in rural areas, carry out comprehensive renovations of coal-fired boilers, and increase the elimination of small coal-fired boilers. In our results, we found that after 2012, the upward trend in the period RRs due to APE slowed down. Related studies have also found that the decrease in household solid fuel consumption has been responsible for most of the reductions in indoor PM_2.5_ pollution in China, and the solid fuel consumption of the whole society, which mainly comes from power, industrial, and transportation sources, is responsible for the ambient PM_2.5_ pollution [23]. The cohort RRs attributable to PM _2.5_ fluctuated relative to the rates for the 1945 reference cohort. Similar to the period RRs, the changes in the cohort RRs were opposites for APE and HPE. The cohort RRs indicate that the risk of lung cancer due to APE is higher than that due to HPE for younger generations. Compared with adults, children are more sensitive to ambient PM_2.5_ exposure due to their small airways and immature detoxification and metabolic systems [24]. To prevent children from being affected by PM_2.5_ for an extended time, we should pay attention to protection against long-term chronic damage [25]. The net drift attributable to PM_2.5_ was below 0, which shows that the impact of PM_2.5_ on lung cancer has declined in China, but the extent of the decline was small. The net drift values for APE and HPE are mainly due to the impact of increased APE hazards.

Although GBD 2019 provided sufficient data that contained age- and sex-specific all-cause and cause-specific indicators, there were some limitations in our study’s ability to reduce the possibility of misclassifying outcomes. First, GBD 2019 could collect missing data and improve its data quality and comparability by modifying and adjusting its data sources and collection and evaluation methods, but it still cannot eliminate bias, which affects the accuracy of the results. Second, the APC model only takes the effects of age, period, and cohort into account and does not further analyse other risk factors.

Our study is the first to analyse the age-period-cohort effects in the temporal trends in lung cancer mortality attributable to PM_2.5_ exposure and to focus on a comprehensive comparison of APE and HPE. Our study found that in terms of PM_2.5_ exposure, the effect of APE on the lung cancer burden is higher than that of HPE, and PM_2.5_ exposure is more harmful to males and older people. The WHO has recommended that public policies and interventions can improve air quality and consequently can have wide-ranging health benefits. Based on the above findings, it is necessary to implement public policies and interventions to reduce the effects of APE in the next few years in order to achieve the goal of reducing the burden of lung cancer.

## Supplementary Information

**Additional file 1: Supplementary 1.** The steps in searching data on the online GBD tool. **Supplementary 2.** The definitions of APC model parameters. **Supplementary 3.** The wald x^2^ test of age, period and cohort effects.

## Data Availability

All our research data are obtained from GBD 2019, the website was http://ghdx.healthdata.org/ gbd-results-tool.
