# Supplementary material for: The epidemiological trends in the burden of lung cancer attributable to PM2.5 exposure in China
Source: BMC Public Health. 2021 Apr 15;21:737. doi: 10.1186/s12889-021-10765-1 (PMC8051098; doi:10.1186/s12889-021-10765-1)
Supplement: Supplementary file 1 — Additional file 1: Supplementary 1. The steps in searching data on the online GBD tool. Supplementary 2. The definitions of APC model parameters. Supplementary 3. The wald x2 test of age, period and cohort effects. [file 12889_2021_10765_MOESM1_ESM.docx]

**The epidemiological trends in the burden of lung cancer attributable to PM_2.5_ exposure in China**

**Running title:** The burden of lung cancer attributable to PM_2.5_ exposure in China

Xiaomei Wu^1^, Bo Zhu^2^*, Jin Zhou^3^, Yifei Bi^4^, Shuang Xu^5^, Baosen Zhou^1^*

1. Department of Clinical Epidemiology and Center of Evidence Based Medicine, The First Hospital of China Medical University, Shenyang, Liaoning Province, China, E-mail: wxm_555555@sina.com

2. Department of Cancer Prevention and Treatment, Cancer Hospital of China Medical University/Liaoning Cancer Hospital & Institute, Shenyang, Liaoning Province, China, E-mail:15998896991@163.com

3. Department of Medical Oncology, Cancer Hospital of China Medical University/Liaoning Cancer Hospital & Institute, Shenyang, Liaoning Province, China, E-mail: zhoujin126@126.com

4. College of Foreign Languages, University of Shanghai for Science and Technology, Shanghai, China, E-mail: biyifei320@gmail.com

5. Library of China Medical University, Shenyang, Liaoning Province, China, E-mail: [sxu@cmu.edu.cn](mailto:sxu@cmu.edu.cn)

*Corresponding author:

Bo Zhu, Department of Cancer Prevention and Treatment, Cancer Hospital of China Medical University/Liaoning Cancer Hospital & Institute, No. 44 Xiaoheyan Road, Dadong District Shenyang, Liaoning Province, 110001, China.

E-mail:15998896991@163.com.

Baosen Zhou, Department of Clinical Epidemiology and Center of Evidence Based Medicine, The First Hospital of China Medical University, No. 155 Nanjing Bei Street, Heping District, Shenyang, Liaoning Province, 110001, China.

E-mail: bszhou@cmu.edu.cn.

**Supplementary materials**

**Supplementary 1. The steps in searching data on the online GBD tool**

1. Go to GBD database ( the website: <http://ghdx.healthdata.org/gbd-results-tool>).
2. Specify our query parameters using the tool’s controls. The figure was the GBD Results Tool user interface.

In our studies, the measure was defined as deaths, location was defined as China; Age was defined as 1-4 age-group, 5-9 age-group, 10-14 age-group, 15-19 age-group, ……. 80-84 age-group and 85 plus age-group; Sex was defined as both, male and female. Year was defined as 1990 to 2019. Metric was defined as rate. Cause was defined as tracheal, bronchus, and lung cancer (Tracheal, bronchus, and lung cancer were called lung cancer in our manuscript).

Risk was respectively defined as particulate matter pollution, ambient particulate matter pollution (APE) and Household air pollution from solid fuels (HPE). In GBD 2019, particulate matter pollution includes both APE and HPE. APE is defined as annual average daily exposure to outdoor air concentrations of PM_2.5_, and HPE is defined as individual exposure to PM_2.5_ due to the use of solid cooking fuel. Therefore, particulate matter pollution in GBD 2019 is PM_2.5_ exposure.

1. After all spaces are selected, and download relevant data.

**Supplementary 2. The definitions of APC model parameters**

The longitudinal age curve indicates the fitted longitudinal age-specific rates in reference cohorts adjusted for period deviations.

The period RR is the period relative risk adjusted for age and nonlinear cohort effects in a period versus the reference one.

The cohort RR is the cohort relative risk adjusted for age and nonlinear period effects in a cohort versus the reference one.

Net drift is the overall log-linear trend by calendar period and birth cohort, and indicates the overall annual percentage change.

Local drifts is the log-linear trend by calendar period and birth cohort for each age group to indicate annual percentage changes for each age group.

**Supplementary 3. The wald x^2^ test of age, period and cohort effects.**

|  | **PM2.5 exposure** | | |  | **APE** | | |  | **HPE** | | |
| --- | --- | --- | --- | --- | --- | --- | --- | --- | --- | --- | --- |
|  | X^2^ | df | P-Value |  | X^2^ | df | P-Value |  | X^2^ | df | P-Value |
| **Both sexes** |  |  |  |  |  |  |  |  |  |  |  |
| NetDrift = 0 | 75.93 | 1 | <0.001 |  | 2353.79 | 1 | <0.001 |  | 4837.67 | 1 | <0.001 |
| All Age Deviations = 0 | 2376.06 | 11 | <0.001 |  | 2907.54 | 11 | <0.001 |  | 1881.58 | 11 | <0.001 |
| All Period Deviations = 0 | 52.67 | 4 | <0.001 |  | 451.92 | 4 | <0.001 |  | 313.26 | 4 | <0.001 |
| All Cohort Deviations = 0 | 792.24 | 16 | <0.001 |  | 751.36 | 16 | <0.001 |  | 598.09 | 16 | <0.001 |
| All Period RR = 1 | 149.52 | 5 | <0.001 |  | 2492.03 | 5 | <0.001 |  | 5162.29 | 5 | <0.001 |
| All Cohort RR = 1 | 804.46 | 17 | <0.001 |  | 7330.68 | 17 | <0.001 |  | 10688.63 | 17 | <0.001 |
| All Local Drifts = Net Drift | 786.76 | 13 | <0.001 |  | 743.16 | 13 | <0.001 |  | 594.06 | 13 | <0.001 |
| **Male** |  |  |  |  |  |  |  |  |  |  |  |
| NetDrift = 0 | 31.50 | 1 | <0.001 |  | 1835.73 | 1 | <0.001 |  | 2159.14 | 1 | <0.001 |
| All Age Deviations = 0 | 1956.44 | 11 | <0.001 |  | 1987.21 | 11 | <0.001 |  | 470.84 | 11 | <0.001 |
| All Period Deviations = 0 | 59.76 | 4 | <0.001 |  | 396.31 | 4 | <0.001 |  | 141.59 | 4 | <0.001 |
| All Cohort Deviations = 0 | 615.24 | 16 | <0.001 |  | 525.18 | 16 | <0.001 |  | 277.05 | 16 | <0.001 |
| All Period RR = 1 | 103.33 | 5 | <0.001 |  | 1974.74 | 5 | <0.001 |  | 2316.70 | 5 | <0.001 |
| All Cohort RR = 1 | 640.28 | 17 | <0.001 |  | 5829.25 | 17 | <0.001 |  | 3922.19 | 17 | <0.001 |
| All Local Drifts = Net Drift | 606.18 | 13 | <0.001 |  | 510.56 | 13 | <0.001 |  | 273.74 | 13 | <0.001 |
| **Female** |  |  |  |  |  |  |  |  |  |  |  |
| NetDrift = 0 | 92.768 | 1 | <0.001 |  | 1327.91 | 1 | <0.001 |  | 5282.41 | 1 | <0.001 |
| All Age Deviations = 0 | 585.20 | 11 | <0.001 |  | 765.19 | 11 | <0.001 |  | 2200.89 | 11 | <0.001 |
| All Period Deviations = 0 | 20.47 | 4 | <0.001 |  | 201.81 | 4 | <0.001 |  | 355.66 | 4 | <0.001 |
| All Cohort Deviations = 0 | 375.02 | 16 | <0.001 |  | 351.75 | 16 | <0.001 |  | 540.17 | 16 | <0.001 |
| All Period RR = 1 | 130.73 | 5 | <0.001 |  | 1378.05 | 5 | <0.001 |  | 5638.05 | 5 | <0.001 |
| All Cohort RR = 1 | 376.53 | 17 | <0.001 |  | 3661.16 | 17 | <0.001 |  | 13425.60 | 17 | <0.001 |
| All Local Drifts = Net Drift | 371.623 | 13 | <0.001 |  | 348.21 | 13 | <0.001 |  | 535.66 | 13 | <0.001 |
